# Reassessing the demand for community-based health insurance in rural Senegal: Geographic distance and awareness

**DOI:** 10.1016/j.ssmph.2021.100974

**Published:** 2021-11-19

**Authors:** Marwân-al-Qays Bousmah, Sylvie Boyer, Richard Lalou, Bruno Ventelou

**Affiliations:** a Université de Paris, IRD, INSERM, Ceped, F-75006 Paris, France; b INSERM, IRD, SESSTIM, Sciences Economiques & Sociales de La Santé & Traitement de L’Information Médicale, Aix-Marseille University, Marseille, France; c Université de Paris, MERIT, IRD, F-75006, Paris, France; d Aix Marseille Univ, CNRS, AMSE, Marseille, 13001, France

**Keywords:** Health insurance, Awareness, Geographic distance, Risk preference, Selection bias, Senegal, Sub-Saharan Africa

## Abstract

Limited access to information is one of the main health insurance market imperfections in developing countries. Differential access to information may determine individuals’ awareness of health insurance schemes, thereby influencing their probability of enrollment. Relying on primary data collected in 2019–2020 in rural Senegal, we estimate the uptake of community-based health insurance using a Heckman-type model to correct for awareness-based sample selection bias. Besides showing that health insurance awareness is a precondition for effective enrollment in community-based health insurance schemes, we also bring new evidence on the roles which geographic factors and individual risk preference play in health insurance uptake by rural dwellers. We show that geographic distance prevents individuals from accessing information on health insurance schemes, and discourage those who are informed from enrolling, because of the additional distance they must travel to benefit from covered healthcare services. Results also show that individual risk preference influences health insurance uptake, but only when information barriers are taken into account. Overall, our results could help decision-makers better shape the universal health coverage roadmap, as policies to improve health insurance awareness differ substantially from policies to improve the features of health insurance schemes.

## 1 Introduction

The expansion of health insurance is viewed as a core strategy for achieving universal health coverage (Savedoff et al., 2012). In rural areas in developing countries, this strategy has often been implemented through the creation of community-based health insurance (CBHI) systems. This type of coverage is based on small organizations with voluntary enrollment, sometimes with premiums partly subsidized.

The current literature estimating the determinants of CBHI uptake based on survey data in West Africa mostly relies on the underlying assumption that individuals are perfectly informed about existing health insurance schemes, their principles and their features. However, health insurance markets are subject to asymmetric information and information frictions (Handel et al., 2019), resulting in different levels of knowledge and awareness of health insurance schemes, which in turn affects participation. Qualitative studies in rural West Africa highlighted heterogeneity in people's knowledge of health insurance schemes (De Allegri, Sanon, Bridges, et al., 2006) and indicated that improving knowledge of CBHI schemes was a necessary but not sufficient condition to foster enrollment (De Allegri, Sanon, & Sauerborn, 2006).

In this paper, we address the issue of sample selection bias due to differential CBHI awareness in rural West Africa, such that the effective uptake of CBHI schemes is likely to be conditioned by individuals’ awareness of the existence, principles, and features of these schemes. To our knowledge, this issue has not been previously addressed in the literature estimating health insurance uptake and its determinants. Besides showing that correcting for awareness-based sample selection bias is necessary when estimating the determinants of CBHI uptake, this paper also contributes to the literature by bringing new evidence on the roles which geographic factors and individual risk preference play in CBHI uptake by rural dwellers. Our analysis relies on primary data collected in 2019–2020 in a rural area in Senegal.

## 2 Context: community-based health insurance schemes in rural Senegal

To move towards universal health coverage, the Senegalese government established a policy in 2013 to ensure the implementation of at least one non-profit CBHI in each rural community throughout the country (Daff et al., 2020). Depending on the type of care received, either 50 or 80% of beneficiaries' healthcare costs are covered. Two conditions are required from beneficiaries to be actually covered by the insurance: (1) before accessing care, they first need to obtain a so-called "letter of guarantee" (*lettre de garantie* in French) at their CBHI, which is a technique of control designed to ensure that only actual beneficiaries are covered, and (2) they can only seek care at local healthcare facilities (often only one) which have an agreement with the CBHI. In the presence of large geographic distances to the CBHI organization or to the affiliated health facility, these specificities are not negligible.

Overall, Senegal still has low health insurance enrollment rates (Daff et al., 2020). According to our data, 7% of households in the rural area of Niakhar have at least one member covered by a voluntary health insurance scheme. Furthermore, we estimate an income poverty rate of 49% and an incidence of catastrophic health expenditures of 6% in the area.

## 3 Methods and data

### 3.1 Data: the CMUtuelleS survey

The CMUtuelleS cross-sectional survey was conducted between November 2019 and March 2020 in the rural community of Niakhar (Fatick region, Senegal) (Delaunay et al., 2013) to investigate various dimensions of universal health coverage in Senegal. Stratified based on the health insurance status of their members, 1002 households were surveyed, representing approximately one-third of all households in this area of 30 villages. The study population for the present analysis includes 1607 adults aged 15 years and older.^1^ A description of the survey design is provided in the Supplementary Material (Appendix A2).

**Fig. 1:**
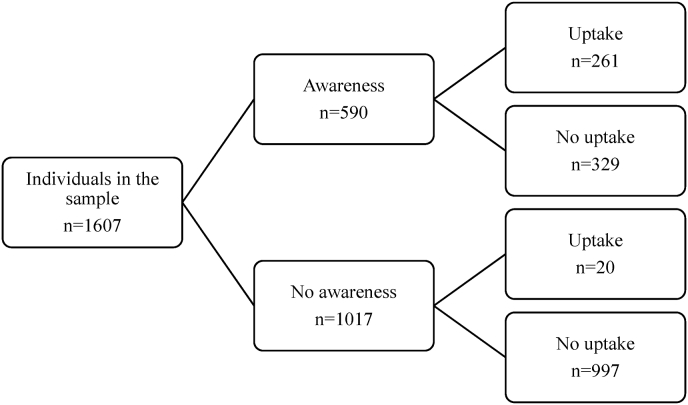
Data structure: awareness and uptake of community-based health insurance.

The main outcome variable is *CBHI uptake* (binary self-reported status). The selection variable is *CBHI awareness*, which captures individuals' knowledge of the existence, principles, and features of CBHI schemes. Namely, awareness is defined as having at least a "fair" knowledge of existing CBHI schemes. The standardized interviewing procedure to assess CBHI awareness is provided in the Supplementary Material (Appendix A3). Fig. 1 suggests that CBHI awareness is a strong precondition for effective uptake.^2^

The 203 km^2^ area of Niakhar has four main health facilities; inhabitants may enroll in one of the two CBHI in the area, depending on the location of their village. Based on GPS coordinates, we compute two different geographic distance variables: (1) the distance (in km) to the nearest CBHI, and (2) the *differential distance* (in km) between a healthcare-seeking journey of an unenrolled patient (defined as the distance to the nearest health facility) and that of a CBHI-enrolled patient, who has to travel an additional distance to obtain a "letter of guarantee" and afterwards has to seek care in a facility which the CBHI have an agreement with. We include a measure of individual risk preference, introduced by Dohmen et al. (2011), namely a qualitative scale ranging from 0 (“not at all willing to take risks”) to 10 (“very willing to take risks”). Other variables include the log of equivalized household consumption expenditure, self-assessed health, sex, marital status, age, age squared, and formal education level. The definitions and summary statistics of all variables used are provided in the Supplementary Material (Appendix A4).

### 3.2 Econometric model

Existing empirical estimations of the demand for health insurance based on survey data in West Africa mostly rely on a simple probit model. However, differential awareness of health insurance schemes is likely to affect CBHI uptake. Failures to address this common issue of sample selection could result in biased and inconsistent estimates (Heckman, 1979). We therefore use the following corrective method for sample selection proposed by Van de Ven and Van Praag (1981) for the case of probit models, which is analogous to Heckman's (1979) method:

(1) $$y_{i}^{*}=x_{i}\beta +\varepsilon_{1i}$$

(2) $$z_{i}=\left(v_{i}\gamma +\varepsilon_{2i}>0\right)$$

(3) $$y_{i}=\left\{ \begin{array}{l} 1\;\\ 0 \end{array}\right.\;\begin{array}{l} y_{i}^{*}>0,\;z_{i}>0\\ otherwise \end{array}$$

where $\varepsilon_{1}∼N\left(0,1\right)$ and $\varepsilon_{2}∼N\left(0,1\right)$ and $corr\left(\varepsilon_{1},\varepsilon_{2}\right)=\rho$.

In the latent Equation (1), $y_{i}^{*}$ is an unobservable latent variable for the conditional probability of CBHI uptake of individual i, $x_{i}$ is a vector of variables affecting individual i’s decision to enroll, and $\varepsilon_{1}$ is an error term. In the selection Equation (2), $z_{i}$ is a binary variable for whether the individual i is aware of the existing health insurance schemes, $v_{i}$ is a vector of variables affecting individual i’s awareness of CBHI schemes, and $\varepsilon_{2}$ is an error term. The error structure allows correlation between $\varepsilon_{1}$ and $\varepsilon_{2}$. The outcome Equation (3) is the probability of enrollment in a CBHI scheme, which is observed only for the sub-sample of individuals aware of the schemes. Also note that $x_{i}$ is contained in $v_{i}$ to avoid producing potentially inconsistent estimators of the β (Vella, 1998; Wooldridge, 2010).

Based on economic intuition and statistical properties, formal education level and the distance to the nearest CBHI are included in the awareness equation only. Regarding formal education, we thus assume that, when also controlling for the wealth effect, the residual effect of formal education on actual health insurance uptake only runs through CBHI awareness. From a statistical standpoint, formal education level satisfies the exclusion restriction condition: it has no effect on the uptake of CBHI other than its influence through CBHI awareness.^3^ Regarding the distance variables, we assume that (simple) remoteness from the CBHI impedes the uptake of health insurance only through its influence on the level of knowledge of CBHI, while the differential distance (the additional travel distance to obtain a "letter of guarantee" and then visit an affiliated health facility) dampens both CBHI awareness and uptake. This is "logical," although we recognize that it may be considered as a weak exclusion restriction since a significant correlation between the distance to the nearest CBHI and actual enrollment among CBHI-aware individuals remains (ρ=−0.153,p<0.001), which could be linked to the fact that the two distances are not independent of each other.

The model is estimated via maximum likelihood. Standard errors are clustered at the household level to account for intra-household correlation. Regressions are weighted using sampling weights to account for choice-based stratified samples and are performed using the *heckprobit* command in Stata (StataCorp, 2015).

Although our primary interest is to investigate the CBHI uptake decision process, we also conduct robustness analyses considering those individuals who either (1) have been enrolled in a CBHI without their knowledge up to the time of the survey (14 individuals), or (2) are enrolled in a CBHI scheme but have no knowledge of their principles and features (6 individuals). This may indeed question the use of CBHI awareness as a selection variable rather than a "simple" determinant of CBHI uptake. First, we estimate the sample selection model including arbitrarily those individuals in the sub-sample of CBHI-aware individuals. Second, we derive a simultaneous-equation model of CBHI awareness and uptake that we estimate on the whole sample. These analyses are fully described in the Supplementary Material (Appendix A6).

## 4 Results

Regression results of the main analysis are presented in Table 1. Results of a simple probit model of CBHI uptake - the usual choice in existing studies on health insurance demand based on survey data in West Africa - are presented for comparison purposes. In the bivariate probit model with sample selection based on awareness of CBHI schemes, the Wald test of independent equations indicates that we can reject the null hypothesis that the selection equation and the CBHI uptake equation are independent. The measured correlation in the errors of the two equations is significantly different from zero (Rho=−0.408,p<0.01), validating the assumption that CBHI awareness is a precondition for effective enrollment in CBHI schemes.

**Table 1:** Regression results.

	**Simple probit**	**Bivariate probit with sample selection**			
	**Uptake of CBHI (coefficient estimates)**	**Awareness of CBHI (coefficient estimates)**	**Marginal effects on the predicted probability of selection**	**Uptake of CBHI (coefficient estimates)**	**Marginal effects on the predicted probability of uptake conditional on selection**
**Distance to the nearest CBHI (in km)**	−0.087*** (0.02)	−0.057*** (0.02)	−0.015*** (0.00)		
**Differential distance (enrolled - unenrolled) (in km)**	−0.052** (0.03)	−0.065*** (0.02)	−0.018*** (0.01)	−0.039 (0.03)	−0.013** (0.01)
**Equivalized household consumption expenditure (in log)**	0.346^∗∗∗^ (0.09)	0.127 (0.10)	0.034 (0.03)	0.375^∗∗∗^ (0.14)	0.093^∗∗∗^ (0.03)
**Individual risk tolerance**	0.002 (0.01)	0.083^∗∗∗^ (0.02)	0.022^∗∗∗^ (0.00)	−0.068^∗∗∗^ (0.02)	−0.009^∗^ (0.00)
**Self-assessed health (ref.=Better health)**					
Poorer health	0.158^∗∗^ (0.08)	0.421^∗∗∗^ (0.09)	0.110^∗∗∗^ (0.02)	−0.200 (0.13)	−0.015 (0.03)
**Sex x Marital status (ref.=Man x In a union)**					
Man x Not in a union	0.079 (0.31)	0.169 (0.39)	Woman:−0.094^∗∗∗^ (0.02) Union:0.040 (0.06)	−0.117 (0.36)	Woman: 0.049^∗∗^ (0.02) Union:−0.003 (0.04)
Woman x Not in a union	0.181 (0.14)	−0.225 (0.15)	Union: 0.040 (0.06)	0.303 (0.27)	Union: −0.003 (0.04)
Woman x In a union	−0.066 (0.07)	−0.341^∗∗∗^ (0.09)		0.320^∗∗^ (0.13)	
**Age**	−0.070^∗∗∗^ (0.01)	0.011 (0.02)	Age:−0.003^∗∗∗^ (0.00)	−0.096^∗∗∗^ (0.03)	Age:−0.001 (0.00)
**Age squared**	0.001^∗∗∗^ (0.00)	−0.000 (0.00)		0.001^∗∗∗^ (0.00)	
**Level of formal education (ref.=None)**					
Primary	0.363^∗∗∗^ (0.10)	0.533^∗∗∗^ (0.11)	0.163^∗∗∗^ (0.04)		
Middle school	0.805^∗∗∗^ (0.16)	1.436^∗∗∗^ (0.21)	0.481^∗∗∗^ (0.07)		
High school or higher	1.094^∗∗∗^ (0.22)	2.015^∗∗∗^ (0.37)	0.640^∗∗∗^ (0.08)		
**Constant**	−3.056^∗∗∗^ (1.03)	−2.228^∗∗^ (1.10)		−1.644 (1.61)	
**Model statistics**					
**No. of observations**	1607	1607		590	
**Wald Chi**^**2**^**(df)**	166.16 (13)	48.74 (9)			
Prob > Chi^2^	0.0000	0.0000			
**Rho**		−0.408			
Wald test (Rho = 0): Chi^2^ (1)		8.04			
Prob > Chi^2^		0.0046			

Notes: ^∗^ p < 0.1, ^∗∗^ p < 0.05, ^∗∗∗^ p < 0.01. Robust standard errors (clustered at the household level to account for intra-household correlation) in parenthesis. Regressions are weighted using sampling weights to account for choice-based stratified samples. The Delta method is applied to calculate the statistical significance of the marginal effects.

The discussion below focuses on the marginal effects on the probability of awareness for the selection equation, and on the marginal effects on the predicted probability of CBHI uptake conditional on selection for the main outcome equation. Conditional marginal effects represent the percentage point change in the CBHI-aware individuals’ predicted probability of enrollment.

A 1 km increase in distance from the nearest CBHI reduces the probability of awareness by 1.5 percentage points. Distance exerts an additional constraint by reducing the probability of CBHI awareness and uptake by 1.8 and 1.3 percentage points, respectively, when the differential distance increases by 1 km. Note that the estimate of the differential distance on uptake is estimated after having accounted for the first negative effect of geographic distances on awareness (and thereby uptake). It is also worth mentioning that we are in the presence of large distances to the nearest CBHI organization (with a median of 5.0 km) and large differential distances (with a median of 2.7 km).

Wealth is positively associated with CBHI uptake. Interestingly, individual risk preference is associated with CBHI uptake only when addressing the issue of selection (no significant association is found in the simple probit model). First, the more individuals are willing to take risks, the higher their probability of being aware of CBHI schemes. Then, after considering the information barrier, risk tolerance is negatively associated with the probability of actual enrolment in a CBHI. Moreover, poorer self-assessed health increases the probability of being aware of available CBHI schemes by 11 percentage points, but does not further influence the probability of enrollment in aware individuals. Age has an (expected) negative relationship with awareness of CBHI schemes, and then exhibits a U-shaped relationship with CBHI-aware individuals’ enrollment decision. Finally, there is a marked positive gradient between formal education level and awareness of available CBHI schemes.

Results of the robustness analyses considering the particular case of CBHI beneficiaries who are not aware of health insurance schemes are provided in the Supplementary Material (Appendix A6). Overall, the results suggest considering CBHI awareness as a selection variable rather than a "simple" determinant of CBHI uptake. A series of other robustness checks are provided in Appendix A7, in which we: (1) considered an alternative definition of the selection variable, (2) test the null hypothesis of independent equations at each step of a backward stepwise process, and (3) test for multicollinearity.

## 5 Discussion

### 5.1 The role of geographic factors

Our model suggests that geographic distance impedes progress towards universal health coverage in two ways: first it reduces individuals' opportunities to access information on available CBHI schemes; second it discourages those who are informed from enrolling because in order to receive CBHI-covered healthcare, the beneficiary must travel an additional distance to obtain a "letter of guarantee" and to go to a health facility which has an agreement with the CBHI.

Relying only on Euclidean straight-line distances is a limitation of the study. However, we could not consider other distance metrics (such as road network distances) and account for different forms of heterogeneity (such as road quality and season-dependent road accessibility), which would have allowed us to be more confident in our analysis. Nevertheless, we believe that our distance variables are good proxies for geographic accessibility, for two main reasons. First, a recent study on spatial mobility within the Niakhar area identified three dominant centers - the villages of Diohine, Ngayokhème, and Toucar (Ndonky et al., 2021) - where most of the health facilities and CBHI organizations of the area are located. A network of unpaved roads directly connects the neighboring non-dominant centers to the dominant centers. We thus believe that the bias of relying on straight-line distances should be minimized compared with other settings.^4^ Second, our data indeed shows a marked positive correlation between the (Euclidean straight-line) distance to the nearest health facility and a self-assessed measure of difficulty to reach the health facility to seek care (ρ=0.386,p<0.001).

Two policy implications can be drawn from the analysis. First, in terms of improving public awareness, information campaigns about CBHI schemes and their benefits should be intensified, especially in the most remote areas. Second, densifying the territorial network of CBHI could increase awareness and in turn health insurance uptake, while also simplifying the healthcare seeking journey of the CBHI-patient (by relaxing the "letter of guarantee" system and increasing the number of CBHI-affiliated health structures) would further increase uptake among CBHI-aware individuals. The latter point assumes that the role of the two distances (direct and differential) is indeed causal, although we cannot rule out the possibility that distance is simply a correlate of other unobserved characteristics (e.g., social network peer effects). The impacts of such policies may be investigated in future research projects, by using randomized control trials (e.g., randomly removing the requirement to obtain a "letter of guarantee" at the CBHI) or by implementing more feasible research designs, for instance using a realist evaluation approach (Pawson & Tilley, 1997) when it comes to densifying the CBHI network.

### 5.2 Risk preference and the demand for health insurance

Our findings also help clarify the role of risk preference in CBHI uptake. In the literature, while Bonan et al. (2014) showed that risk-averse heads of households had a higher willingness to pay for health microinsurance in Senegal, Bonan et al. (2017) found that risk aversion did not influence uptake. Dercon et al. (2019) showed that, surprisingly, risk aversion may be negatively associated with health insurance demand in settings where trust in insurance schemes is limited (due to misperceptions of product attributes). Li et al. (2021) investigated the influence of households' stated risk preferences on their health insurance enrollment and portfolio allocation decisions in China, showing that the lower the insured households’ risk aversion, the greater their probability of owning risky assets. However, they did not find this risk substitution between medical expenditure risk and financial risk for households with high risk aversion.

Our model for rural Senegal shows that risk preference does indeed play a role on health insurance enrolment decisions, but only when the distortions related to the provision of information are accounted for. Specifically, risk tolerance is first associated with higher health insurance awareness, a result which could be linked to the role of trust in the insurer highlighted by Dercon et al. (2019). Then, a positive relationship between risk aversion and the uptake of health insurance is only exhibited in individuals sufficiently well informed about available health insurance schemes, while no significant association between risk preference and CBHI uptake was found in the simple probit model of CBHI uptake estimated on the whole sample. This is an important study result as it suggests that the (theoretically) expected process behind a rational CBHI enrollment decision does indeed take place, but only when the issue of selection based on health insurance awareness has properly been treated.

## 6 Conclusions

When assessing the demand for health insurance in rural settings in developing countries, our study highlights the need to take one of the main distortions in the provision of health insurance into account, specifically limited access to information about available schemes. In turn, this leads to differential awareness of these schemes. Our results provide evidence of sample selection bias in the estimation of the demand for health insurance, which is shown to be preconditioned by individuals’ awareness of the existence, principles, and features of CBHI schemes. Failure to correct for such bias would distort the estimates of the determinants of CBHI uptake.

Although we cannot rule out the possibility that the estimated effects may be partly driven by unobserved confounding factors, preventing us from estimating causal effects, we are able to disentangle the factors influencing CBHI awareness from those influencing uptake, while correcting for the selection bias resulting from the differential awareness in the estimation of the health insurance demand. Our findings also contribute to the literature by investigating the role of geographic factors and risk preference more closely.

Overall, our results could help decision-makers better shape the universal health coverage roadmap, as policies to improve access to information and health insurance awareness differ substantially from policies to improve the features of health insurance schemes.

## Funding

This research is part of the UNISSAHEL program (Universal Health Coverage in Sahel), funded by the 10.13039/501100011061Agence Française de Développement (AFD).

## Availability of data and material

Data are available from the authors upon reasonable request (contact: Marwân-al-Qays Bousmah. CEPED (UMR 196), Université de Paris, Campus Saint-Germain, 45 Rue des Saints-Pères, 75,006 Paris, France. Tel.: +33643521166. E-mail: marwan-al-qays.bousmah@ird.fr).

## Code availability

Available from the authors upon reasonable request.

## Author statement

BV led the UNISSAHEL economic research program (WP4). MQB performed the econometric analysis. The manuscript was drafted by MQB, SB, RL and BV. All authors made critical comments on the manuscript and agreed to be responsible for all aspects of the work.

## Ethical statement

The CMUtuelleS survey was approved by the Senegalese National Ethical Committee for Health Research (n°000037/MSAS/DPRS/CNERS and n°0000118/MSAS/DPRS/CNERS). Informed consent was obtained from all subjects.

## Declaration of competing interest

All authors report no conflict of interest in relationship with this study.

## Footnotes

1 Individuals whose enrollment in a CBHI was assigned and fully subsidized by the government through the so-called *Bourse de Sécurité Familiale* program were excluded from the analysis, as they did not have to make any enrollment decision.

2 Note that 20 beneficiaries are not aware of CBHI schemes (namely, they do not have an at least fair knowledge of existing CBHI schemes). These individuals, who are by definition not included in the awareness-based selected sample, either (1) know the existence of CBHI schemes but have no knowledge of their principles and features, or (2) have been enrolled in a CBHI without their knowledge up to the time of the survey.

3 When also included in the uptake equation, formal education is not statistically significant while remaining significant in the awareness equation, as shown in Appendix A5 in the Supplementary Material.

4 The rural area of Niakhar, which includes 30 villages connected by a network of unpaved roads, is located in the continental Sahelian-Sudanese climatic zone. Our data on a sub-sample of 211 individuals who sought care at a health facility within the past two months before the survey indicate that the most commonly used modes of transport are horse or donkey carts (49.8%), followed by walking (38.9%) and by car (16.6%), the other modes of transports representing less than 1% of the journeys. The area has a mainly flat topography (with a maximum elevation of 20 m), with zones potentially flooded during the rainy season (Delaunay et al., 2013; Faye et al., 2020; Ndonky et al., 2021). Thus, mobility conditions tend to be easier in the dry rather than in the rainy season (Ndonky et al., 2021). Investigating the effects of such heterogeneity - which is not captured in our study - is an interesting avenue for future research.
