# Supplementary material for: Reassessing the demand for community-based health insurance in rural Senegal: Geographic distance and awareness
Source: SSM Popul Health. 2021 Nov 19;16:100974. doi: 10.1016/j.ssmph.2021.100974 (PMC8608590; doi:10.1016/j.ssmph.2021.100974)
Supplement: Multimedia component 1 [file mmc1.docx]

**Appendix A. Supplementary material**

This online resource has been provided by the authors to give readers additional information about their work.

Supplement to: *Reassessing the* *demand for community-based health insurance in rural Senegal: geographic distance and awareness*.

Contents:

Page 2: Appendix A1. Members of the UNISSAHEL Study Group

Page 3: Appendix A2. The CMUtuelleS survey

Page 4: Appendix A3. Assessment of awareness of community-based health insurance (CBHI)

Page 5: Appendix A4. Definitions and summary statistics of the dependent and independent variables

Page 6: Appendix A5. Test of validity of formal education level as exclusion restriction

Page 7-10: Appendix A6. Robustness checks: CBHI-awareness as a selection variable?

Page 11-14: Appendix A7. Other robustness checks

Page 15: References

**Appendix A1. Members of the UNISSAHEL Study Group**

**Project coordination:** MP. BA (UGB), E. BONNET (RESILIENCE, IRD), F. CHABROL (CEPED, IRD), A. DESGREES DU LOU (CEPED, IRD), A. DUMONT (CEPED, IRD), F. EBOKO (CEPED, IRD), A. FILLOL (CEPED, IRD), D. MANOUFI (BASE), V. RIDDE (CEPED, IRD), K. SOW (CRCF), L. TOURE (MISELI), B. VENTELOU (AMSE, CNRS).

**Project members:** Mohammad ABU ZAINEH (AMSE), Malick Ousman AHMAT (BASE), Sameera AWAWDA (AMSE, IRD), El Hadj BA (VITROME, IRD), Pauline BOIVIN (MISELI), Marwân-al-Qays BOUSMAH (CEPED, IRD), Sylvie BOYER (SESSTIM, Aix-Marseille Université), Valérie DELAUNAY (LPED, IRD), Fatimatou DIA (UGB), Mariama DIEDHIOU (CRCF), Rama Sao DIOP (UGB), Fatou DIOP (CRCF), Cheikh Mouhamadou Falilou Mb. FALL (UGB), Laurence FLEURY (LPED, IRD), Idriss Ali Zakaria GALI-GALI (BASE), Lara GAUTIER (UDEM), Wendyam Charles Paulin Didier KABORE (CEPED, IRD), Yamba KAFANDO (independent consultant), Richard LALOU (MERIT, IRD), Ramadan KAGONTA (BASE), Gabrièle LABORDE-BALEN (TRANSVIHMI, IRD), Fatou Bintou NIANG (UGB), Ratnan NGADOUM (Université de N’Djamena), Khady Seck NGOM (CRCF), El Hadji Ibrahima Kady SARR (UGB), Mathieu SEPPEY (UDEM), Cheikh SOKHNA (VITROME, IRD), Souleymane SOW (CRCF), Bernard TAVERNE (TRANSVIHMI, IRD), Cheikh TINE (MERIT, IRD), Grâce-à-Dieu TOULAO (IRD), Bintou RASSOUL TOP (CRCF), Mamadou YATOUDEME (IRD).

**Acronyms:** AMSE (France): Aix-Marseille School of Economics, CEPED (France): Centre Population et Développement, CNRS (France): Centre National de la Recherche Scientifique, CRCF (Senegal): Centre Régional de Recherche et de Formation à la Prise en Charge Clinique de Fann, LASDEL (Niger): Laboratoire d'Etudes et de Recherche sur les Dynamiques Sociales et le Développement Local, LPED (France): Laboratoire Population Environnement Développement, SESSTIM (France): Sciences économiques et sociales de la santé et traitement de l’information médicale, UDEM (Canada): Université de Montréal, UGB (Sénégal): Université Gaston Berger de Saint Louis.

**Appendix A2. The CMUtuelleS survey**

Due to the low health insurance enrollment rate in rural Senegal (Daff et al., 2020), in order to have sufficient statistical power, a stratified survey was preferred over a general population survey. We conducted a preliminary study to identify individuals enrolled in a CBHI, merging the individuals’ identifiers from the CBHI registers into the Niakhar Health and Demographic Surveillance System (Delaunay et al., 2013).

Households were then stratified into three groups: (A) households with at least one voluntary insured individual, (B) households with at least one individual insured through the BSF national program (and no voluntary insured individuals), and (C) households with neither voluntary nor subsidized insured individuals. Group (A) households were selected exhaustively due to their relatively small number (n=255), while households in the two other groups were randomly selected to have 300 households in each group (representing approximately 30% and 20% of the population in group (B) and (C), respectively).

A household-level questionnaire was administered to the head of the household, or to the most knowledgeable proxy respondent in the household if the head was missing. Then, an adult-level questionnaire was administered to up to two adults (≥ 15 years) in the household. In group (A) households, the main CBHI-enrolled member was selected (the other potential CBHI-enrolled members being beneficiaries) and, if in a union, her/his partner. In group (B), the main CBHI-enrolled adult was selected (*i.e*, the one designated to receive the BSF cash transfer) and, if in a union, her/his partner. In group (C), the household head was selected and, if in a union, her/his partner.

The household-level questionnaire gathered information on socioeconomic and demographic characteristics, detailed expenditures, and financial risk exposition and coping strategies. The adult-level questionnaire gathered information on socioeconomic and demographic characteristics, economic activity, health and quality of life, healthcare utilization, health insurance, individual preferences, and perceptions of healthcare quality.

The final sample included 1,002 households and 1,787 adults. More households have been randomly selected to meet the objective of 300 households in group (B) and (C), as the repartition of households in each group slightly changed between the preliminary study and the survey. The survey was then matched with the Niakhar Health and Demographic Surveillance System to benefit from additional data on households’ and individuals’ socio-demographic characteristics (*e.g.*, GPS coordinates).

**Appendix A3. Assessment of awareness of community-based health insurance (CBHI)**

This assessment was made by the interviewer based on a standardized interviewing procedure about the knowledge of CBHI existence, principles, and features. First, the respondent was asked whether he/she had ever heard of CBHI organizations. If yes, the respondent was asked to describe the main principles and features of the CBHI scheme, with the possibility to complete his/her description in case of incomplete answers. The interviewer was asked to use three levels of knowledge that were clearly predefined: poor, fair, and good. Poor knowledge was defined as knowing the existence of CBHI schemes, but not their main principles and features. Fair knowledge was defined as mentioning that a non-refundable insurance premium has to be payed, and that in case of illness the healthcare costs are partly covered. Good knowledge was defined as mentioning supplementary information such as the amount of the insurance premium, the CBHI organizations that exist in the area, the voluntary characteristic of membership, the principle of solidarity of CBHI schemes. Finally, the variable of awareness of existing CBHI schemes distinguishes individuals with and without an at least fair knowledge of CBHI schemes.

**Appendix A4. Definitions and summary statistics of the dependent and independent variables**

| **Variable** | **Type** | **Definition** | **Mean or proportion** | **Standard deviation** | **Min** | **Max** |
| --- | --- | --- | --- | --- | --- | --- |
| Awareness of CBHI | Binary | Without at least a fair knowledge of CBHI schemes (=reference category); With at least a fair knowledge | 0.25 | 0.43 | 0 | 1 |
| Uptake of CBHI | Binary | Not enrolled in a CBHI (=reference category); Enrolled | 0.05 | 0.21 | 0 | 1 |
| Distance to the nearest CBHI | Continuous | Shortest geographical distance (in km) between the household and the CBHI (based on GPS coordinates) | 5.53 | 2.84 | 0.06 | 12.82 |
| Differential distance (enrolled - unenrolled) | Continuous | Differential distance (in km) between a healthcare-seeking journey of an unenrolled patient (defined as the distance to the nearest health facility) and that of a CBHI-enrolled patient (defined as the distance to the nearest CBHI to obtain a ‘letter of guarantee,’ plus the additional distance to the health facility which the CBHI have an agreement with) (based on GPS coordinates) | 2.53 | 2.27 | 0.00 | 7.22 |
| Equivalized household consumption expenditure | Continuous | Log of total monthly consumption expenditures (in CFA Francs) per consumption equivalent in the household | 10.50 | 0.45 | 8.84 | 12.20 |
| Individual risk tolerance | Discrete | Qualitative scale ranging from 0 (“not at all willing to take risks”) to 10 (“very willing to take risks”) | 5.19 | 2.49 | 0 | 10 |
| Self-assessed health | Binary | Excellent/Very good (=reference category); Poorer health (Good/Fair/Poor) | 0.59 | 0.49 | 0 | 1 |
| Sex | Binary | Man (=reference category); Woman | 0.53 | 0.50 | 0 | 1 |
| Marital status | Binary | In a union (=reference category); Not in a union | 0.91 | 0.28 | 0 | 1 |
| Age | Continuous | In years | 52.85 | 14.00 | 14 | 94 |
| Level of formal education | Categorical | None (=reference category); Primary; Middle school; High school or higher | 0.22 | 0.56 | 0 | 3 |
| Notes: n=1607. Data are weighted using sampling weights to account for choice-based stratified samples. | | | | | | |

**Appendix A5. Test of validity of formal education level as exclusion restriction**

|  | **Bivariate probit with sample selection** | |
| --- | --- | --- |
|  | **Awareness of CBHI**  **(coefficient estimates)** | **Uptake of CBHI**  **(coefficient estimates)** |
| **Distance to the nearest CBHI (in km)** | -0.059***  (0.02) |  |
| **Differential distance (enrolled - unenrolled) (in km)** | -0.063***  (0.02) | 0.004  (0.04) |
| **Equivalized household consumption expenditure (in log)** | 0.122  (0.10) | 0.271  (0.17) |
| **Individual risk tolerance** | 0.083***  (0.02) | -0.079***  (0.02) |
| **Self-assessed health (ref.=Better health)**  Poorer health | 0.417***  (0.09) | -0.302**  (0.14) |
| **Sex x Marital status (ref.=Man x In a union)**  Man x Not in a union  Woman x Not in a union  Woman x In a union | 0.186  (0.39)  -0.224  (0.15)  -0.342***  (0.09) | -0.091  (0.36)  0.327  (0.25)  0.365***  (0.12) |
| **Age** | 0.011  (0.02) | -0.086***  (0.03) |
| **Age squared** | -0.000  (0.00) | 0.001***  (0.00) |
| **Level of formal education (ref.=None)**  Primary  Middle school  High school or higher | 0.540***  (0.11)  1.434***  (0.21)  1.982***  (0.37) | -0.236  (0.19)  -0.498  (0.37)  -0.408  (0.45) |
| **Constant** | -2.191**  (1.09) | -0.265  (1.95) |
| **Model statistics** |  | |
| No. of observations | 1,607 | 590 |
| Wald Chi^2^ (df)  Prob > Chi^2^ | 54.78 (12)  0.0000 | |
| Rho  Wald test (Rho = 0): Chi^2^ (1)  Prob > Chi^2^ | -0.710  4.32  0.0377 | |
| Notes: ^∗^ p < 0.1, ^∗∗^ p < 0.05, ^∗∗∗^ p < 0.01. Robust standard errors (clustered at the household level to account for intra-household correlation) in parenthesis. Regressions are weighted using sampling weights to account for choice-based stratified samples. The Delta method is applied to calculate the statistical significance of the marginal effects. | | |

**Appendix A6. Robustness checks: CBHI-awareness as a selection variable?**

Although our primary interest is to investigate the CBHI uptake decision process, we conduct robustness analyses considering those 20 individuals who either (1) have been enrolled in a CBHI without their knowledge up to the time of the survey (14 individuals), or (2) are enrolled in a CBHI scheme but have no knowledge of their principles and features (6 individuals). These 20 individuals may indeed question the use of CBHI awareness as a selection variable rather than a ‘simple’ determinant of CBHI uptake. We conduct two robustness analyses to address this concern.

First, we arbitrarily include those 20 individuals in the sub-sample of CBHI-aware individuals, considering them as ‘false’ CBHI-unaware individuals instead of ‘false’ CBHI-beneficiaries. The results, which remain qualitatively similar to those from the primary analysis, are provided in Table A6.1 below.

**Table A6.1. Bivariate probit with sample selection, arbitrarily including the 20 beneficiaries who are not aware of CBHI schemes in the sub-sample of CBHI-aware individuals: regression results**

|  | **Bivariate probit with sample selection** | | | |
| --- | --- | --- | --- | --- |
|  | **Awareness of CBHI**  **(coefficient estimates)** | **Marginal effects on the predicted probability of selection** | **Uptake of CBHI**  **(coefficient estimates)** | **Marginal effects on the predicted probability of uptake conditional on selection** |
| **Distance to the nearest CBHI (in km)** | -0.058***  (0.02) | -0.016***  (0.00) |  |  |
| **Differential distance (enrolled - unenrolled) (in km)** | -0.065***  (0.02) | -0.018***  (0.01) | -0.037  (0.03) | -0.013**  (0.01) |
| **Equivalized household consumption expenditure (in log)** | 0.125  (0.10) | 0.034  (0.03) | 0.343**  (0.14) | 0.090***  (0.03) |
| **Individual risk tolerance** | 0.081***  (0.02) | 0.022***  (0.00) | -0.074***  (0.02) | -0.012**  (0.01) |
| **Self-assessed health (ref.=Better health)**  Poorer health | 0.424***  (0.09) | 0.112***  (0.02) | -0.199  (0.13) | -0.018  (0.03) |
| **Sex x Marital status (ref.=Man x In a union)**  Man x Not in a union  Woman x Not in a union  Woman x In a union | 0.201  (0.39)  -0.210  (0.15)  -0.344***  (0.09) | Woman:  -0.096***  (0.02)  Union:  0.048  (0.06) | -0.119  (0.35)  0.349  (0.27)  0.301**  (0.12) | Woman: 0.051**  (0.02)  Union:  0.007  (0.05) |
| **Age** | 0.009  (0.02) | Age:  -0.003***  (0.00) | -0.095***  (0.03) | Age:  -0.001  (0.00) |
| **Age squared** | -0.000  (0.00) |  | 0.001***  (0.00) |  |
| **Level of formal education (ref.=None)**  Primary  Middle school  High school or higher | 0.545***  (0.11)  1.421***  (0.21)  1.990***  (0.37) | 0.168***  (0.04)  0.477***  (0.07)  0.634***  (0.08) |  |  |
| **Constant** | -2.131*  (1.09) |  | -1.317  (1.60) |  |
| **Model statistics** |  | | | |
| No. of observations | 1,607 |  | 610 |  |
| Wald Chi^2^ (df)  Prob > Chi^2^ | 47.35 (9)  0.0000 | | | |
| Rho  Wald test (Rho = 0): Chi^2^ (1)  Prob > Chi^2^ | -0.373  6.47  0.0110 | | | |
| Notes: ^∗^ p < 0.1, ^∗∗^ p < 0.05, ^∗∗∗^ p < 0.01. Robust standard errors (clustered at the household level to account for intra-household correlation) in parenthesis. Regressions are weighted using sampling weights to account for choice-based stratified samples. The Delta method is applied to calculate the statistical significance of the marginal effects. | | | | |

Second, we derive a simultaneous-equation model of CBHI awareness and uptake that we estimate on the whole sample (n=1,607). More specifically, we jointly model CBHI awareness and uptake as two probit equations, with CBHI awareness introduced in latent form on right-hand side of the uptake equation. To do so, we consider the following mixed-process simultaneous system:

| $\Pr\left( \text{CBHI awareness}_{i}=1 \right)=\Phi(\gamma+{\theta X}_{i}+{\lambda Z}_{i}+\psi_{i})$ | (A6.1) |
| --- | --- |
| $\Pr\left( \text{CBHI uptake}_{i}=1 \right)=\Phi(\sigma+\beta\text{CBHI awareness}_{i}^{\boldsymbol{\dagger}}+{\varphi X}_{i}+\mu_{i})$ | (A6.2) |

where the response variable is CBHI awareness (binary) and uptake (binary) in equation (A6.1) and (A6.2), respectively. $X$ includes all the exogenous variables common to both equations, and $Z$ includes the exclusion restrictions that allow identification of the model (namely, the formal education level and the distance to the nearest CBHI). The error structure allows correlation between the two error terms $\psi$ and $\mu$. CBHI awareness appears on the right-hand side of the uptake equation (A6.2) and is modeled as latent (not fully observed). As in the primary analysis, all regressions are weighted using sampling weights to account for choice-based stratified samples. Standard errors are clustered at the household level to account for intra-household correlation. This full information maximum likelihood (FIML) estimation is performed using Stata (StataCorp, 2019) and the *cmp* command (Roodman, 2011).

Structural estimates are presented in Table A6.2 below. As expected, the importance of the (latent) linear predictors of CBHI awareness in determining uptake is assessed. Also importantly, the estimated correlation coefficient between the two equations is not significantly different from zero ($atanhrho=0.145;p=0.340$), indicating that estimating the two equations separately (and on the whole sample) would not yield biased and inconsistent estimates due to simultaneity bias. Sample selection bias may nevertheless be present, an issue which is specifically addressed in the primary analysis of the paper.

**Table A6.2. Simultaneous-equation system estimated on the whole sample: regression results**

|  | **Simultaneous-equation model** | |
| --- | --- | --- |
|  | **Awareness of CBHI** | **Uptake of CBHI** |
|  | **Probit**  **(structural estimates)** | **Probit**  **(structural estimates)** |
| **Awareness of CBHI^†^** |  | 0.994***  (0.17) |
| **Distance to the nearest CBHI (in km)** | -0.057***  (0.02) |  |
| **Differential distance (enrolled - unenrolled) (in km)** | -0.064**  (0.03) | -0.065**  (0.03) |
| **Equivalized household consumption expenditure (in log)** | 0.135  (0.10) | 0.335**  (0.14) |
| **Individual risk tolerance** | 0.082***  (0.02) | -0.074***  (0.03) |
| **Self-assessed health (ref.=Better health)**  Poorer health | 0.429***  (0.09) | -0.145  (0.14) |
| **Sex x Marital status (ref.=Man x In a union)**  Man x Not in a union  Woman x Not in a union  Woman x In a union | 0.119  (0.37)  -0.225  (0.15)  -0.337***  (0.09) | -0.006  (0.49)  0.412  (0.26)  0.253*  (0.14) |
| **Age** | 0.009  (0.02) | -0.099***  (0.03) |
| **Age squared** | -0.000  (0.00) | 0.001***  (0.00) |
| **Level of formal education (ref.=None)**  Primary  Middle school  High school or higher | 0.541***  (0.11)  1.445***  (0.20)  1.918***  (0.31) |  |
| **Constant** | -2.283**  (1.11) | -2.540  (1.63) |
| **Model statistics** |  | |
| No. of observations | 1,607 | |
| Log pseudolikelihood | -3744.3851 | |
| Wald Chi^2^ (df)  Prob > Chi^2^ | 231.01 (22)  0.0000 | |
| Atanhrho | 0.145  (0.15) | |
| Notes: Robust standard errors (clustered at the household level to account for intra-household correlation) in parenthesis. Regressions are weighted using sampling weights to account for choice-based stratified samples. Atanhrho is the arc-hyperbolic tangent of the correlation coefficient.  ^∗^ p < 0.1, ^∗∗^ p < 0.05, ^∗∗∗^ p < 0.01.  **^†^** In latent form. | | |

**Appendix A7. Other robustness checks**

We consider an alternative definition of the selection variable, which (simply) distinguishes individuals with and without knowledge of the existence of CBHI schemes (irrespective of their knowledge of CBHI principles and features). Results are presented in Table A7.1 below. As expected, the use of the Heckman procedure to correct for sample selection bias is justified, as we reject the null hypothesis of independent equations ($Rho=-0.623;p<0.001$). The main assumption guiding our research strategy holds.

**Table A7.1. Bivariate probit with sample selection, considering a larger definition of CBHI awareness (that is, having knowledge of the existence of CBHI schemes): regression results**

|  | **Bivariate probit with sample selection** | | | |
| --- | --- | --- | --- | --- |
|  | **Awareness of CBHI**  **(coefficient estimates)** | **Marginal effects on the predicted probability of selection** | **Uptake of CBHI**  **(coefficient estimates)** | **Marginal effects on the predicted probability of uptake conditional on selection** |
| **Distance to the nearest CBHI (in km)** | -0.072***  (0.02) | -0.023***  (0.01) |  |  |
| **Differential distance (enrolled - unenrolled) (in km)** | -0.036  (0.02) | -0.012  (0.01) | -0.031  (0.03) | -0.010**  (0.00) |
| **Equivalized household consumption expenditure (in log)** | 0.253***  (0.10) | 0.080***  (0.03) | 0.151  (0.13) | 0.054***  (0.02) |
| **Individual risk tolerance** | 0.051***  (0.02) | 0.016***  (0.01) | -0.040**  (0.02) | -0.003  (0.00) |
| **Self-assessed health (ref.=Better health)**  Poorer health | 0.524***  (0.09) | 0.162***  (0.03) | -0.285**  (0.11) | -0.007  (0.02) |
| **Sex x Marital status (ref.=Man x In a union)**  Man x Not in a union  Woman x Not in a union  Woman x In a union | 0.022  (0.39)  -0.353**  (0.15)  -0.367***  (0.08) | Woman:  -0.118***  (0.03)  Union:  0.006  (0.07) | 0.075  (0.36)  0.485**  (0.23)  0.288***  (0.10) | Woman: 0.025  (0.02)  Union:  0.034  (0.04) |
| **Age** | 0.007  (0.02) | Age:  -0.005***  (0.00) | -0.077***  (0.02) | Age:  -0.001  (0.00) |
| **Age squared** | -0.000  (0.00) |  | 0.001***  (0.00) |  |
| **Level of formal education (ref.=None)**  Primary  Middle school  High school or higher | 0.407***  (0.10)  1.203***  (0.22)  1.683***  (0.38) | 0.139***  (0.03)  0.417***  (0.07)  0.545***  (0.08) |  |  |
| **Constant** | -2.877***  (1.10) |  | 0.045  (1.47) |  |
| **Model statistics** |  | | | |
| No. of observations | 1,607 |  | 709 |  |
| Wald Chi^2^ (df)  Prob > Chi^2^ | 40.03 (9)  0.0000 | | | |
| Rho  Wald test (Rho = 0): Chi^2^ (1)  Prob > Chi^2^ | -0.623  18.85  0.0000 | | | |
| Notes: ^∗^ p < 0.1, ^∗∗^ p < 0.05, ^∗∗∗^ p < 0.01. Robust standard errors (clustered at the household level to account for intra-household correlation) in parenthesis. Regressions are weighted using sampling weights to account for choice-based stratified samples. The Delta method is applied to calculate the statistical significance of the marginal effects. | | | | |

We also test the robustness of the results using a backward stepwise process, with three groups of variables removed sequentially from the both equations (yet always keeping the exclusion restrictions to avoid producing inconsistent estimates). Namely, we first remove self-assessed health and risk tolerance (Table A7.2, Model 1), then wealth (Table A7.2, Model 2), and finally the demographic variables (sex, marital status, and age) (Table A7.2, Model 3). In all estimations, the null hypothesis of independent equations is still rejected ($Rho\neq0$). Note that these estimations are likely to suffer from omitted variable bias.

**Table A7.2. Backward stepwise process**

|  | **Bivariate probit with sample selection** | | | | | |
| --- | --- | --- | --- | --- | --- | --- |
|  | **Model 1** | | **Model 2** | | **Model 3** | |
|  | **Awareness of CBHI**  **(coefficient estimates)** | **Uptake of CBHI**  **(coefficient estimates)** | **Awareness of CBHI**  **(coefficient estimates)** | **Uptake of CBHI**  **(coefficient estimates)** | **Awareness of CBHI**  **(coefficient estimates)** | **Uptake of CBHI**  **(coefficient estimates)** |
| **Distance to the nearest CBHI (in km)** | -0.053***  (0.02) |  | -0.054***  (0.02) |  | -0.051***  (0.02) |  |
| **Differential distance (enrolled - unenrolled) (in km)** | -0.063**  (0.03) | -0.039  (0.03) | -0.061**  (0.03) | -0.036  (0.03) | -0.063**  (0.02) | -0.048*  (0.03) |
| **Equivalized household consumption expenditure (in log)** | 0.093  (0.09) | 0.363***  (0.14) |  |  |  |  |
| **Individual risk tolerance** |  |  |  |  |  |  |
| **Self-assessed health (ref.=Better health)**  Poorer health |  |  |  |  |  |  |
| **Sex x Marital status (ref.=Man x In a union)**  Man x Not in a union  Woman x Not in a union  Woman x In a union | 0.173  (0.37)  -0.243  (0.15)  -0.358***  (0.08) | -0.153  (0.38)  0.357  (0.26)  0.354***  (0.12) | 0.159  (0.37)  -0.251*  (0.15)  -0.356***  (0.08) | -0.197  (0.39)  0.349  (0.26)  0.369***  (0.12) |  |  |
| **Age** | 0.016  (0.02) | -0.101***  (0.03) | 0.015  (0.02) | -0.101***  (0.03) |  |  |
| **Age squared** | -0.000  (0.00) | 0.001***  (0.00) | -0.000  (0.00) | 0.001***  (0.00) |  |  |
| **Level of formal education (ref.=None)**  Primary  Middle school  High school or higher | 0.472***  (0.11)  1.343***  (0.21)  1.841***  (0.35) |  | 0.471***  (0.10)  1.351***  (0.21)  1.851***  (0.34) |  | 0.544***  (0.10)  1.384***  (0.20)  2.011***  (0.35) |  |
| **Constant** | -1.380  (1.07) | -1.814  (1.57) | -0.378  (0.47) | 2.032***  (0.65) | -0.423***  (0.08) | -0.200  (0.16) |
| **Model statistics** |  | | | | | |
| No. of observations | 1,607 | 590 | 1,607 | 590 | 1,607 | 590 |
| Wald Chi^2^ (df)  Prob > Chi^2^ | 40.01 (7)  0.0000 | | 35.70 (6)  0.0000 | | 3.13 (1)  0.0767 | |
| Rho  Wald test (Rho = 0): Chi^2^ (1)  Prob > Chi^2^ | -0.456  9.07  0.0026 | | -0.494  10.72  0.0011 | | -0.521  16.46  0.0000 | |
| Notes: ^∗^ p < 0.1, ^∗∗^ p < 0.05, ^∗∗∗^ p < 0.01. Robust standard errors (clustered at the household level to account for intra-household correlation) in parenthesis. Regressions are weighted using sampling weights to account for choice-based stratified samples. The Delta method is applied to calculate the statistical significance of the marginal effects. | | | | | | |

Finally, we test for multicollinearity by computing the variance inflation factors (VIF) after estimating the selection and uptake equations individually. Results are displayed in Table A7.3. Any of the variables included appear to be a linear combination of other independent variables (defined as a VIF>10), with all VIF<2, except age and its square, which is expected.

**Table A7.3. Test of multicollinearity: variance inflation factors**

|  | **Variance inflation factors** | | |
| --- | --- | --- | --- |
|  | **Equation:**  **Awareness of CBHI**  **(whole sample)** | **Equation:**  **Uptake of CBHI**  **(CBHI-aware sub-sample)** | **Equation:**  **Uptake of CBHI (whole sample)** |
| **Distance to the nearest CBHI (in km)** | 1.74 |  |  |
| **Differential distance (enrolled - unenrolled) (in km)** | 1.77 | 1.02 | 1.01 |
| **Equivalized household consumption expenditure (in log)** | 1.06 | 1.04 | 1.06 |
| **Individual risk tolerance** | 1.07 | 1.07 | 1.07 |
| **Self-assessed health** | 1.13 | 1.14 | 1.13 |
| **Sex x Marital status**  Man x Not in a union  Woman x Not in a union  Woman x In a union | 1.04  1.11  1.33 | 1.04  1.10  1.27 | 1.02  1.10  1.31 |
| **Age** | 40.94 | 35.23 | 40.05 |
| **Age squared** | 40.95 | 35.22 | 40.45 |
| **Level of formal education**  Primary  Middle school  High school or higher | 1.07  1.05  1.05 |  |  |
| **Mean VIF** | 7.33 | 8.68 | 9.80 |
|  | 1,607 | 590 | 1,607 |
| Notes: VIF=variance inflation factors for the independent variables specified in a linear regression model. Regressions are weighted using sampling weights to account for choice-based stratified samples. | | | |

**References**

Daff, B. M., Diouf, S., Diop, E. S. M., Mano, Y., Nakamura, R., Sy, M. M., Tobe, M., Togawa, S., & Ngom, M. (2020). Reforms for financial protection schemes towards universal health coverage, Senegal. *Bulletin of the World Health Organization*, *98*(2), 100‑108. https://doi.org/10.2471/BLT.19.239665

Delaunay, V., Douillot, L., Diallo, A., Dione, D., Trape, J.-F., Medianikov, O., Raoult, D., & Sokhna, C. (2013). Profile : The Niakhar Health and Demographic Surveillance System. *International Journal of Epidemiology*, *42*(4), 1002‑1011. https://doi.org/10.1093/ije/dyt100

Roodman, D. (2011). Fitting fully observed recursive mixed-process models with cmp. *The Stata Journal*, *11*(2), 159‑206. https://doi.org/10.1177/1536867X1101100202

StataCorp. (2019). *Stata statistical software : Release, 16*. StataCorp LP.
